# *Odontochilus putaoensis* (Cranichideae, Orchidaceae), a new species from Myanmar

**DOI:** 10.3897/phytokeys.103.25913

**Published:** 2018-07-02

**Authors:** Ye Lwin Aung, Aye Thin Mu, Xiaohua Jin

**Affiliations:** 1 State Key Laboratory of Systematic and Evolutionary Botany, Institute of Botany, Chinese Academy of Sciences, Beijing 100093, China; 2 Southeast Asia Biodiversity Research Institute, Chinese Academy of Sciences, Yezin, Nay Pyi Taw 05282, Myanmar

**Keywords:** Cranichideae, Kachin State, key, new species, southeast Asia, terrestrial orchid

## Abstract

*Odontochilus putaoensis*, a new species of Orchidaceae, is described and illustrated from Putao Township, Kachin State, Myanmar. *Odontochilus putaoensis* is close to *O. duplex*, but can be easily distinguished from the latter by having a light yellow lip, a bisaccate hypochile with a small, erect, blade-like and emarginate callus within each sac, a mesochile with a pair of dentate-pectinate flanges and a bilobed epichile with a pair of widely diverging lobes that are erect and concave. An identification key to the Southeast Asian species of *Odontochilus* and colour photographs of *O. putaoensis* are provided. A preliminary conservation assessment according to the IUCN Red List Categories and Criteria is given for the new species.

## Introduction

*Odontochilus* Blume (1858) (Orchidaceae, Orchidoideae, Cranichideae) consists of approximately 40 species, distributed from tropical Asia, Pacific islands to Japan, subtropical mainland Asia and eastern Himalayas (Pridgeon et al. 2003, Chen et al. 2009, Pedersen et al. 2011, Chase et al. 2015, Tang et al. 2016). Most species of *Odontochilus* are small terrestrial plants, usually found in humid evergreen broadleaved forests. The generic delimitation of *Odontochilus* has been confused for a long time with its relative *Anoectochilus* Blume (1825). As both genera share some floral characters such as pectinate mesochile, ventral column wings and two stigma lobes, they were usually considered as one genus (Lang 1999, Pridgeon et al. 2003). Morphologically, *Odontochilus* is distinguished from *Anoectochilus* by its saccate and non-extruded spur enclosed by the lateral sepals and two parallel stigma lobes positioned under the rostellum, whereas *Anoectochilus* has a conical spur extruded beyond the lateral sepals and two remote stigma lobes (Lin and Hsu 1976, Pridgeon et al. 2003, Chen et al. 2009, Pedersen et al. 2011). Recent results of molecular systematics indicated that *Odontochilus* is closely related to *Chamaegastrodia* and *Rhomboda*, whereas *Anoectochilus* is closely related to *Ludisia* (Li et al. 2016).

In the continental part of southeast Asia, there are nine species of *Odontochilus* (Seidenfaden 1992, Schuiteman et al. 2008, Chen et al. 2009, Pedersen et al. 2011, Kurzweil and Lwin 2014, Averyanov et al. 2015, Tang et al. 2016). Although there is no recorded species of *Odontochilus* in the checklist of Kress et al. (2003), there are several species listed as occurring in Myanmar in the floristic documents of Chen et al. (2009), Pedersen et al. (2011) and Kurzweil and Lwin (2014). During our fieldwork in Putao Township, Kachin State, northern Myanmar, in October 2014, a new species of *Odontochilus* was discovered and is described below.

## Material and methods

All measurements of the new *Odontochilus* species were taken from dried herbarium specimens and field notes. In the description, length and width are represented as length × width. In total, four living plants and one dried specimen of the new species were examined. All measurements of *O. duplex* (Holttum) Ormerod (Peninsular Thailand and Peninsular Malaysia) were based on literature (Seidenfaden and Wood 1992, Ormerod 2005, Pedersen et al. 2011).

## Taxonomic treatment

### Odontochilus putaoensis

Taxon classificationPlantaeAsparagalesOdontochilus

X.H. Jin, L.A. Ye & A.T. Mu sp. nov.

urn:lsid:ipni.org:names:77186066-1

Figure 1

#### Diagnosis.

*Odontochilus putaoensis* is similar to *O. duplex*, but can be easily distinguished from the latter by having a light yellow lip composed of a bisaccate hypochile with a small, erect, blade-like and emarginate callus within each sac, a mesochile with a pair of dentate-pectinate flanges and bilobed epichile with a pair of widely diverging lobes that are erect and concave.

#### Type.

MYANMAR. Kachin State: Putao Township, Hponkanrazi Wildlife Sanctuary, subtropical, evergreen, broad-leaved, montane forest, 2000 m a.s.l., 20 October 2014, *Xiaohua Jin et al, PT-ET 959* (Holotype, PE!).

#### Description.

Plants autotrophic, terrestrial, 40–60 cm tall. Stem ascending, pubescent, 2–6-leaved. Leaves dark green, ovate-lanceolate, 6–7.5 × 3.2–4 cm, attenuate at apex, blade glabrous; petiole-like base and tubular sheath ca. 2.8 cm long. Peduncle pubescent, with 1 or 2 sheathing bracts, reddish-brown, ovate-lanceolate, 12–15 × 4–5 mm, pubescent, long acuminate; rachis pubescent, sub-densely 16-flowered; floral bracts reddish–brown, ovate-lanceolate, ca. 9 × 4 mm, as long as ovary, abaxially pubescent, long acuminate at apex. Flowers resupinate; dorsal sepal forming a hood with petals, dark greenish-brown, ovate, ca. 6 × 3 mm, acute at apex, abaxially pubescent; lateral sepals greenish-brown, elliptic, oblique, ca. 8 × 4 mm, acute at apex, abaxially pubescent. Petals obliquely ovate-falcate, ca. 6 × 3 mm, membranous, glabrous; lip light yellow, T-shaped, shallowly grooved along the mid-line, ca. 1 cm long; hypochile bisaccate, sac sub-globose, ca. 2 mm in diameter, containing a low median keel and a small, erect, blade-like and apically emarginate callus on each side; mesochile ca. 4 mm long, with a pair of dentate-pectinate flanges, each flange composed of three narrow filaments, ca. 4 mm long and one broad blade-like posterior filament, ca. 3 mm long; epichile bilobed, lobes erect, diverging at obtuse angle to each other, elliptic, ca. 5 × 3 mm, margin involute and consequently resulting in concave lobes, obtuse at apex. Column ca. 1 mm long, stout; anther acuminate in front, ca. 4 mm long; pollinia 2, clavate; stigma lobes confluent; ovary and pedicel cylindric, twisted, sparsely pubescent.

**Figure 1.**
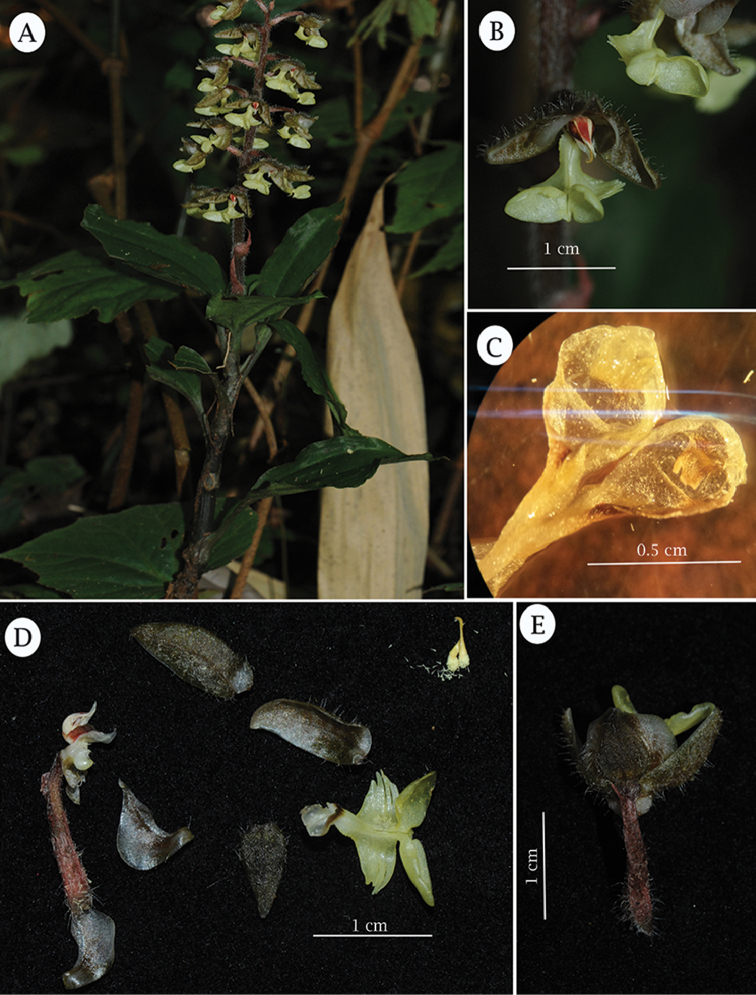
*Odontochilus putaoensis* X.H.Jin, L.A.Ye & A.T.Mu. **A** Habit of *Odontochilus putaoensis* **B** Front view of flower, showing lip epichile with a pair of erect and concave lobes **C** Hypochile of *Odontochilus putaoensis*, indicating small, erect, blade-like, emarginate callus within each sac **D** Dissected flower, showing pedicel and ovary, column, sepals, petals, lip and a pair of clavate pollinia **E** Dorsal view of flower, showing dorsal sepal forming a hood with petals. Photographed by X.H. Jin.

#### Etymology.

The new species is named after Putao, the northernmost town of Myanmar, near which it was discovered in a vast area of undisturbed mountain forest.

#### Distribution and habitat.

*Odontochilus putaoensis* grows in shaded and damp humus in humid, broad-leaved, evergreen forest, at an elevation of about 1500-2000 m. At present, *O. putaoensis* is only known from the type locality.

#### Conservation status.

**Least Concern (LC).** *Odontochilus putaoensis* was collected in the Hponkanrazi Wildlife Sanctuary, Putao Township, Kachin State, northern Myanmar. Until now, only one population, consisting of ca. 200 individuals, has been discovered in the vast reserve of 2704 km^2^. As there is no threat currently affecting the quality of its habitat and there is also a considerable number of mature individuals, the species is here preliminarily assigned a status of Least Concern (LC) according to the guidelines for using the IUCN Red List Categories and Criteria (IUCN Standards and Petitions Subcommittee, 2017).

### Key to *Odontochilus* in the continental part of southeast Asia

**Table:** 

1	Plant without green leaves, all leaves reduced to sheaths. Flowers usually not resupinate	***O. poilanei***
1'	Plant with green leaves, leaves fully differentiated. Flowers usually resupinate	
2	Epichile not deeply bilobed, broadly obovate to transversely oblong	
3	Mesochile with a pair of entire flanges (or slightly dentate), epichile nearly as wide as long	***O. macranthus***
3'	Mesochile with a pair of dentate flanges (or slightly dentate), epichile about twice as wide as long	***O. uniflorus***
2'	Epichile deeply bilobed	
4	Mesochile with two pairs of laciniate flanges	***O. duplex***
4	Mesochile with one pair of dentate-pectinate flanges	
5	Ovary usually glabrous	
6	Bracts finely erose-ciliate, flowers usually yellow	***O. lanceolatus***
6'	Bracts entire, flowers usually white	***O. brevistylis***
5'	Ovary (glandular-) pubescent, sometimes sparsely so	
7	Epichile bilobed with a pair of widely diverging lobules that are erect and concave	***O. putaoensis***
7'	Epichile bilobed with a pair of diverging and flat lobules	
8	Leaves reddish-brown, labellum twisted	***O. tortus***
8'	Leaves green above, labellum not twisted	***O. elwesii***

## Discussion

Myanmar lies in southeast Asia and is well endowed with biodiversity-rich areas such as tropical evergreen rainforest, coastal mangrove forest and subtropical montane forest. The northern part of Myanmar is situated in the ecological transition area of three global biodiversity hotspots, the Indo-Burma hotspot, Mountains of southwest China hotspot and Himalaya hotspot (Myers et al. 2000, Sodhi et al. 2004, Mittermeier et al. 2011, Khine et al. 2017, Jin et al. 2018). However, the biodiversity of northern Myanmar is far less understood due to the absence of scientific research. Recently, biodiversity research activities in northern Myanmar have been jointly conducted in cooperation with international research institutions, resulting in discoveries of new species of fauna and flora, such as *Aristolochia sinoburmanica* Y.H.Tan & B.Yang, *Bulbophyllum putaoensis* Q.Liu, *Coelogyne putaoensis* X.H.Jin, L.A.Ye & Schuit, *Gastrodia kachinensis* X.H.Jin & L.A.Ye, *G. putaoensis* X.H.Jin, *Hedychium putaoense* Y.H.Tan & H.B.Ding, *Kerivoula kachinensis*, *Muntiacus putaoensis*, *Oreoglanis hponkanensis*, *Rhinopithecus strykeri*, *Selliguea kachinensis* Hovenkamp, S.Linds. & Fraser-Jenk. and so on (Amato et al. 1999, Bates et al. 2004, Geissmann et al. 2011, Khine et al. 2016, Aung et al. 2017, Chen et al. 2017, Jin and Kyaw 2017, Liu et al. 2017, Aung and Jin 2018, Ding et al. 2018, Yang et al. 2018).

*Odontochilus putaoensis* is a very distinctive species in having easily identifiable floral features such as a pair of erect and concave epichile lobes. Having such distinctive floral features, *O. putaoensis* can be easily distinguished from its closely related species, *O. duplex*, although both species are more or less similar in their vegetative as well as floral characters. In addition, their altitudinal range and habitat type are relatively comparable: *O. putaoensis* was collected at 1500–2000 m elevation and *O. duplex* at ca. 750 m elevation (Pedersen et al. 2011). *Odontochilus putaoensis* grows in subtropical broad-leaved, evergreen forest in northern Myanmar, whereas *O. duplex* in the upper tropical rainforest in Peninsular Thailand and Peninsular Malaysia (Pedersen et al. 2011). As well, *O. putaoensis* flowers in October while *O. duplex* in May (Pedersen et al. 2011).

## Supplementary Material

XML Treatment for Odontochilus putaoensis

## References

[B1] Amato G Egan MG Rabinowitz A (1999) A new species of muntjac, *Muntiacus putaoensis* (Artiodactyla: Cervidae) from northern Myanmar. Animal Conservation 2(1): 1–7. https://doi.org/10.1111/j.1469-1795.1999.tb00042.x

[B2] Aung YL Jin XH Schuiteman A (2017) *Coelogyne putaoensis* (Orchidaceae), a new species from Myanmar. PhytoKeys 82: 27–34. https://doi.org/10.3897/phytokeys.82.13172 10.3897/phytokeys.82.13172 PMC5546387 28794680

[B3] Aung YL Jin XH (2018) *Gastrodia kachinensis* (Orchidaceae), a new species from Myanmar. In: Jin XH Shui YM Tan YH Kang M (Eds) Plant diversity in Southeast Asia. PhytoKeys 94: 23–29. https://doi.org/10.3897/phytokeys.94.21348 10.3897/phytokeys.94.21348 PMC5799787 29416417

[B4] Averyanov LV Nguyen KS Tich NT Nguyen PT Nong VD Nguyen VC Xuan CC (2015) New orchids in the flora of Vietnam. Wulfenia 22: 137–188.

[B5] Bates PJJ Struebig MJ Rossiter SJ Kingston T Oo SSL Mya KM (2004) A new species of *Kerivoula* (Chiroptera: Vespertilionidae) from Myanmar (Burma). Acta Chiropterologica 6(2): 219–226. https://doi.org/10.3161/001.006.0203

[B6] Blume CL (1825) Ludwig von Bijdragen tot de flora van Nederlandsch Indië 8: 411. http://dx.doi.org/10.5962/bhl.title.395

[B7] Blume CL (1858) Collection des orchidées les plus remarquables de l’archipel Indien et du Japon. Sulphe, Amsterdam, icons, 360 pp.

[B8] Chase MW Cameron KM Freudenstein JV Pridgeon AM Salazar G van den Berg C Schuiteman A (2015) An updated Classification of Orchidaceae. Botanical Journal of the Linnean Society 177(2): 151–174. https://doi.org/10.1111/boj.12234

[B9] Chen XQ Gale SW Cribb PJ Ormerod P (2009) *Odontochilus*. In: Wu ZY, Raven PH, Hong DY (Eds) Flora of China (Vol. 25). Science Press, Beijing and Missouri Botanical Garden Press, St. Louis, 80‒84.

[B10] Chen XY Qin T Chen ZY (2017) *Oreoglanis hponkanensis*, a new sisorid catfish from north Myanmar (Actinopterygii, Sisoridae). ZooKeys 646: 95–108. https://doi.org/10.3897/zookeys.646.11049 10.3897/zookeys.646.11049 PMC5299442 28228678

[B11] Ding HB Bin Y Zhou SS Li R Maw MB Kyaw WM Tan YH (2018) *Hedychium putaoense* (Zingiberaceae), a new species from Putao, Kachin State, Northern Myanmar. In: Jin XH Shui YM Tan YH Kang M (Eds) Plant diversity in Southeast Asia. PhytoKeys 94: 51–57. https://doi.org/10.3897/phytokeys.94.22065 10.3897/phytokeys.94.22065 PMC5799740 29416420

[B12] Geissmann T Lwin N Aung SS Aung TN Aung ZM Hla TH Grindley M Momberg F (2011) A New Species of Snub-Nosed Monkey, Genus *Rhinopithecus* Milne-Edwards, 1872 (Primates, Colobinae), From Northern Kachin State, Northeastern Myanmar. American Journal of Primatology 73(1): 96–107. https://doi.org/10.1002/ajp.20894 20981682 10.1002/ajp.20894

[B13] Standards IUCN, Petitions Subcommittee (2017) Guidelines for Using the IUCN Red List Categories and Criteria. Version 13. Prepared by the Standards and Petitions Subcommittee. http://www.iucnredlist.org/documents/RedListGuidelines.pdf [accessed: May 30, 2017]

[B14] Jin XH Kyaw M (2017) *Gastrodia putaoensis* sp. nov. (Orchidaceae, Epidendroideae) from North Myanmar. Nordic Journal of Botany 35: 730‒732. https://doi.org/10.1111/njb.01581

[B15] Jin XH Tan YH Quan RC (2018) Taxonomic discoveries bridging the gap between our knowledge and biodiversity. In: Jin XH, Shui YM, Tan YH, Kang M (Eds) Plant diversity in Southeast Asia. PhytoKeys 94: 1‒2. https://doi.org/10.3897/phytokeys.94.23887 10.3897/phytokeys.94.23887 PMC5799726 29416414

[B16] Khine PK Lindsay S Fraser-Jenkins C Kluge J Kyaw M Hovenkamp P (2016) *Selliguea kachinensis* (Polypodiaceae), a new fern species of uncertain affinity from Northern Myanmar. PhytoKeys 62: 73‒81. https://doi.org/10.3897/phytokeys.62.8101 10.3897/phytokeys.62.8101 PMC4856904 27212883

[B17] Khine PK Fraser-Jenkins C Lindsay S Middleton D Miehe G Thomas P Kluge J (2017) A Contribution Toward the Knowledge of Ferns and Lycophytes from Northern and Northwestern Myanmar. American Fern Journal 107(4): 219–256. https://doi.org/10.1640/0002-8444-107.4.219

[B18] Kress J Robert A DeFilippes E Kyi YY (2003) A Checklist of the Trees, Shrubs, Herbs, and Climbers of Myanmar. http://www.botany.si.edu/myanmar [accessed: May 30, 2017]

[B19] Kurzweil H Lwin S (2014) A guide to orchids of Myanmar. Natural History Publications (Borneo), Kota Kinabalu, 196 pp.

[B20] Lang KY (1999) *Odontochilus*. In: Lang KY, Chen SC, Luo YB, Zhu GH (Eds) Flora Reipublicae Popularis Sinicae (Vol. 17). Science Press, Beijing, 205‒227.

[B21] Li MH Zhang GQ Lan SR Liu ZJ, China Phylogeny Consortium (2016) A molecular phylogeny of Chinese orchids. Journal of Systematics and Evolution 54(4): 349‒362. https://doi.org/10.1111/jse.12187

[B22] Lin TP Hsu CC (1976) Orchid Genera, *Anoectochilus* and *Odontochilus* of Taiwan. Taiwania 21(2): 229–236.

[B23] Liu Q Zhou SS Li R Zhang MX Zyaw M Lone S Quan RC (2017) *Bulbophyllum putaoensis* (Orchidaceae: Epidendroideae; Malaxideae), a new species from Kachin State, Myanmar. Phytotaxa 305(1): 57–60. https://doi.org/10.11646/phytotaxa.305.1.9

[B24] Mittermeier RA Turner WR Larsen FW Brooks TM Gascon C (2011) Global biodiversity conservation: the critical role of hotspots. In: Zachos FE Habel JC (Eds) Biodiversity Hotspots. Springer Publishers, London, 3–22.

[B25] Myers N Mittermeier RA Mittermeier CG da Fonseca GAB Kent J (2000) Biodiversity hotspots for conservation priorities. Nature 403(6772): 853–858. https://doi.org/10.1038/35002501 10706275 10.1038/35002501

[B26] Ormerod P (2005) Notulae Goodyerinae (II). Taiwania 50(1): 1–10.

[B27] Pedersen HÆ Kurzweil H Suddee S Cribb PJ (2011) *Odontochilus* In: Santisuk T, Larsen K, Newman M (Eds) Flora of Thailand (Vol. 12, Part 1), Orchidaceae 1 (Cypripedioideae, Orchidoideae, Vanilloideae). Prachachon Co. Ltd. Press, Bangkok, 184‒196.

[B28] Pridgeon AM Cribb PJ Chase MW Rasmussen FN (2003) Genera Orchidacearum, Volume 3. Orchidoideae (Part one). Oxford University Press, Oxford, 126‒129.

[B29] Schuiteman A Bonnet P Svengsuksa B Barthélémy D (2008) An annotated checklist of the Orchidaceae of Laos. Nordic Journal of Botany 26: 257‒316. https://doi.org/10.1111/j.1756-1051.2008.00265.x

[B30] Seidenfaden G (1992) The Orchids of Indochina. Opera Botanica 114: 1–502.

[B31] Seidenfaden G Wood JJ (1992) The Orchids of Peninsular Malaysia and Singapore. The Royal Botanic Gardens, Kew & Botanic Gardens, Singapore and Olsen & Olsen, Fredensborg, 71–76.

[B32] Sodhi NS Koh LP Brook BW Ng PKL (2004) Southeast Asian biodiversity: an impending disaster. Trends in Ecology and Evolution 19(12): 654‒660. https://doi.org/10.1016/j.tree.2004.09.006 10.1016/j.tree.2004.09.006 16701328

[B33] Tang H Feng HZ Huang YF (2016) *Odontochilus napoensis* sp. nov. (Orchidoideae: Orchidaceae) from southwestern Guangxi, China. Nordic Journal of Botany 34: 405‒408. https://doi.org/10.1111/njb.00944

[B34] Yang B Ding HB Zhou SS Zhu X Li R Maw MB Tan YH (2018) *Aristolochia sinoburmanica* (Aristolochiaceae), a new species from north Myanmar. In: Jin XH Shui YM Tan YH Kang M (Eds) Plant diversity in Southeast Asia. PhytoKeys 94: 13–22. https://doi.org/10.3897/phytokeys.94.21557 10.3897/phytokeys.94.21557 PMC5799737 29416416

